# Average Stand Age from Forest Inventory Plots Does Not Describe Historical Fire Regimes in Ponderosa Pine and Mixed-Conifer Forests of Western North America

**DOI:** 10.1371/journal.pone.0147688

**Published:** 2016-05-19

**Authors:** Jens T. Stevens, Hugh D. Safford, Malcolm P. North, Jeremy S. Fried, Andrew N. Gray, Peter M. Brown, Christopher R. Dolanc, Solomon Z. Dobrowski, Donald A. Falk, Calvin A. Farris, Jerry F. Franklin, Peter Z. Fulé, R. Keala Hagmann, Eric E. Knapp, Jay D. Miller, Douglas F. Smith, Thomas W. Swetnam, Alan H. Taylor

**Affiliations:** 1 John Muir Institute of the Environment, University of California, Davis, CA, 95616, United States of America; 2 USDA Forest Service, Pacific Southwest Region, Vallejo, CA, 94592, United States of America; 3 Department of Environmental Science and Policy, University of California, Davis, CA, 95616, United States of America; 4 USDA Forest Service, Pacific Southwest Research Station, Davis, CA, 95616, United States of America; 5 USDA Forest Service, Forest Inventory and Analysis Program, Pacific Northwest Research Station, Portland, OR, 97205, United States of America; 6 USDA Forest Service, Forest Inventory and Analysis Program, Pacific Northwest Research Station, Corvallis, OR, 97331, United States of America; 7 Rocky Mountain Tree-Ring Research, Fort Collins, CO, 80526, United States of America; 8 Biology Department, Mercyhurst University, Erie, PA, 16546, United States of America; 9 Dept. Forest Management, University of Montana, Missoula, MT, 59812, United States of America; 10 School of Natural Resources and the Environment, University of Arizona, Tucson, AZ, 85721, United States of America; 11 Laboratory of Tree-Ring Research, University of Arizona, Tucson, AZ, 85721, United States of America; 12 National Park Service, Pacific West Region, Fire and Aviation Management, Klamath Falls, OR, 97601, United States of America; 13 School of Environmental and Forest Sciences, University of Washington, Seattle, WA, 98195, United States of America; 14 School of Forestry, Northern Arizona University, Flagstaff, AZ, 86011, United States of America; 15 USDA Forest Service, Pacific Southwest Research Station, Redding, CA, 96002, United States of America; 16 USDA Forest Service, Pacific Southwest Region, Fire and Aviation Management, McClellan, CA, 95652, United States of America; 17 Yosemite National Park, Yosemite, CA, 95389, United States of America; 18 Department of Geography and Earth and Environmental Systems Institute, The Pennsylvania State University, University Park, PA, 16802, United States of America; Oregon State University, UNITED STATES

## Abstract

Quantifying historical fire regimes provides important information for managing contemporary forests. Historical fire frequency and severity can be estimated using several methods; each method has strengths and weaknesses and presents challenges for interpretation and verification. Recent efforts to quantify the timing of historical high-severity fire events in forests of western North America have assumed that the “stand age” variable from the US Forest Service Forest Inventory and Analysis (FIA) program reflects the timing of historical high-severity (i.e. stand-replacing) fire in ponderosa pine and mixed-conifer forests. To test this assumption, we re-analyze the dataset used in a previous analysis, and compare information from fire history records with information from co-located FIA plots. We demonstrate that 1) the FIA stand age variable does not reflect the large range of individual tree ages in the FIA plots: older trees comprised more than 10% of pre-stand age basal area in 58% of plots analyzed and more than 30% of pre-stand age basal area in 32% of plots, and 2) recruitment events are not necessarily related to high-severity fire occurrence. Because the FIA stand age variable is estimated from a sample of tree ages within the tree size class containing a plurality of canopy trees in the plot, it does not necessarily include the oldest trees, especially in uneven-aged stands. Thus, the FIA stand age variable does not indicate whether the trees in the predominant size class established in response to severe fire, or established during the absence of fire. FIA stand age was not designed to measure the time since a stand-replacing disturbance. Quantification of historical “mixed-severity” fire regimes must be explicit about the spatial scale of high-severity fire effects, which is not possible using FIA stand age data.

## Introduction

Fire is an integral component of montane coniferous forests of western North America, where complex interactions between topography, vegetation and climate have created a diverse suite of fire regimes and forest structures [1]. Forests in drier areas of this region, particularly ponderosa pine (*Pinus ponderosa* Dougl. ex Laws) and drier mixed-conifer forests, have been greatly altered by logging, grazing, and fire suppression following Euro-American settlement, which has led to decreases in large tree abundance and increases in tree density and fuel loads [2–7]. Such changes have contributed, at least in part, to large contiguous areas of nearly complete canopy mortality from recent large, intense wildfires [8–11]. In an effort to help guide management and restoration efforts for these forests, historical fire regimes are studied to assess how current fire characteristics may have departed from the historical range of variation [7, 12–14].

Extensive evidence from fire scars, forest reconstructions, and early settlement records indicate that many ponderosa pine and drier mixed-conifer forests in western North America burned at frequent (generally <30 year) intervals prior to Euro-American settlement (e.g. [14, 15–18]). When fire burns this frequently, it maintains a heterogeneous uneven-aged forest structure with large fire-resistant trees, tree clumps that escape fire, small gaps, and low or patchy fuel loads that limit the spatial extent of tree mortality, leading to predominantly low- to moderate-severity effects [19–23]. High-severity fire, in contrast, kills most or all overstory trees within a given area, commonly termed “stand replacing fire” [24].

The technique of using forest stand ages to estimate time since last high-severity fire has been used most commonly in boreal-type forests (i.e., high elevation and high latitude conifer forests of the northern hemisphere) with relatively long (>100 year) fire return intervals and characteristically high-severity fire effects that occur across large (>100 ha) spatial areas (e.g., [25, 26–28]). Many tree species in these fire regimes have evolved mechanisms for abundant post-fire seed presence (e.g. serotiny) and rapid dispersal (characteristics found in Rocky Mountain lodgepole pine, *Pinus contorta* var. *latifolia*, jack pine, *P*. *banksiana*, and black spruce, *Picea mariana*) or rapid regeneration via re-spouting from extensive root systems (e.g., trembling aspen, *Populus tremuloides*). In these typically even-aged stands, ages of the dominant cohort of trees may therefore approximate the time since the last high-severity fire, although recruitment periods may lag fire by several decades [25, 29]. Models of fire history based on average age at the stand scale may therefore be appropriate in some boreal or subalpine conifer forests, which are more likely to experience high-severity fire effects that kill the vast majority of canopy trees within a stand or across a landscape [13].

High-severity fire was undoubtedly a component of fire regimes in ponderosa pine and drier mixed-conifer forests [30, 31]. Patches of historical high-severity fire in these forests are not necessarily captured in the fire scar record if all the trees within the patch are killed [32]. As a result, even-aged cohorts of trees in drier mixed-conifer forests have been used as evidence to document the occurrence and spatial scale of historical high-severity patches and/or the approximate time since those events [33–36]. However, in order to infer the rotation of high-severity patches across a landscape, a systematic sample of even-aged stands reflecting the distribution of time since fire would be needed across the entire study area. If plot data are to be treated as a statistical sample of high-severity fire at the landscape level, a strict standard is needed to determine if data from a particular plot represent an even-aged cohort generated by a high-severity fire.

Unlike in boreal-type forests, shorter fire return intervals and the complex, heterogeneous nature of fire spread in ponderosa pine and drier mixed-conifer forests create stands that are distinctly uneven-aged down to the sub-ha scale [15, 19, 37, 38]. Mortality of both overstory and regenerating age classes is incomplete in these forests due to the frequency of fire, and recruitment is spatially heterogeneous and most commonly associated with periodic wet episodes, longer intervals between fires, or general canopy openness both at stand and landscape scales [39–45]. The resulting stands typically defy meaningful description by a single stand age value. One could choose, for example, to define a stand’s age as the age of the oldest live tree, the average age of the most abundant tree-size class, or an average of all canopy tree ages. The results from even these three alternatives would differ markedly. Consequently, time since fire and high-severity fire rotation cannot be meaningfully estimated using forest age structure from these types of forests.

Recently, stand age data from the US Forest Service Forest Inventory and Analysis (FIA) program were interpreted as an indicator of the timing of past high-severity fire events [46]. These data were used to support an argument that high-severity fire in ponderosa pine and mixed-conifer forests of the western United States was much more common and widespread prior to Euro-American settlement than is suggested by reconstructions of fire history and historical stand structure, other types of historical evidence, and modeling studies of fire behavior and fire-vegetation dynamics [2, 4, 7, 19, 33, 47–52]. FIA is a nationwide program that tracks status, change and trend in forest resources based on an extensive network of periodically re-measured sample plots (www.fia.fs.fed.us). The tree inventory area within FIA plots ranges from 0.067–0.4 ha depending on the state and the tree size (25–35 trees measured per plot on average), with one plot per approximately 2400 ha (details in Materials and Methods).

Odion et al. [46] investigated the distribution of the stand age attribute in FIA plot data (hereafter “FIA stand age”) for “unmanaged” forests, and found a greater abundance of plots with FIA stand ages dating to the years just prior to widespread fire suppression (i.e. 1810–1889), relative to the active fire suppression period from 1930–2009 or to the years prior to 1810 ([46]; pg. 7). Because Odion et al. [46] assume that FIA stand age indicates the approximate timing of a stand-replacing disturbance, they interpret this result to mean that the frequency of these events has decreased in the past century. Since this decrease coincides with the period of active fire suppression, they attribute high-severity fire as the primary mechanism “initiating” stands during the 1800’s. Odion et al. [46] contrast this result with their expectation that “unmanaged” forests under a frequent low- to moderate-severity fire regime should be “dominated by older age classes” ([46]; pg. 4).

The conclusion in Odion et al. [46] that high-severity fire was common in the century prior to fire suppression, rests on the central assumption that “the age of relatively young and intermediate-aged stands (e.g. <200 years) reflects the time since a disturbance that shifted dominance from older to younger trees” ([46], pg. 4). This assumption has two corollaries that are required to make the link between FIA stand age and high-severity fire. First, any trees within the FIA plot that are distinctly older than the FIA stand age must necessarily have survived a high-severity fire during the year implied by the FIA stand age. Second, the recruitment of trees roughly the same age as the FIA stand age is only explainable by a high-severity fire event that removed most or all of the pre-existing trees.

In this paper, we examine the validity of this central assumption and its two corollaries, in order to investigate the general question of whether contemporary forest plot data can be used to estimate the time since the last high-severity fire. Specifically, we re-analyzed the FIA data used by Odion et al. [46], in conjunction with additional data linked to known fire history records, to test the following hypotheses: 1) trees older than the FIA stand age should be rare, and when present, should not account for more of the stand’s basal area than would be expected to survive a high-severity fire, and 2) there is minimal evidence of tree recruitment coincident with FIA stand ages in the absence of probable high-severity fire events. We conclude with a discussion of the critical importance of spatial scale of high-severity effects when using the term “mixed-severity fire”.

## Materials and Methods

### FIA stand age

Because the analysis in Odion et al. [46] is based on the “stand age” attribute in the FIA plot network, we briefly describe the methods used to estimate this attribute in the FIA protocol. FIA field crews measure trees within a 0.4-ha plot footprint (for the states of California, Oregon and Washington inventoried by the Pacific Northwest Research Station; hereafter “coastal states”) or a 0.067-ha plot footprint (for the states of Idaho, Montana, Colorado, Wyoming, Nevada, Utah, Arizona and New Mexico inventoried by the Rocky Mountain Research Station; hereafter “interior states”) [53]. For the current analysis and in Odion et al. [46], South Dakota is included among the interior states. Plots are assigned one or more “condition classes” based on distinct changes in vegetation or land use occurring within the plot; many plots have only a single condition class, in which case “condition” is equivalent to “plot”. Each condition class is given a stand age estimate based on the average age of a subset of non-overtopped trees, on or near the plot, selected from the diameter at breast height (dbh) size class that accounts for the plurality of dominant and co-dominant trees in the forest overstory. This predominant size class is termed the “stand-size class”. In coastal states, field crews classify a condition into one of five stand-size classes with minimum dbh values (in conifer forest) of 0 cm, 12.7 cm, 22.9 cm, 50.8 cm, and 101.6 cm (“FLDSZCD” in the FIA database). The distribution of tree diameters and ages from field ring counts of increment cores within the crew-estimated stand-size class is then used to assign a mean stand age in the field. In interior states, algorithms are used to classify a condition into one of three stand-size classes with minimum dbh values of 0 cm, 12.7 cm and 22.9 cm (“STDSZCD” in the FIA database) based on individual tree stocking values, and stand age is calculated as the mean of increment-cored ages in that size class. For example, in coastal states a stand where 50% of the overstory tree canopy cover is associated with trees between 22.9 and 50.8 cm dbh (FLDSZCD = 3), 40% with trees between 50.8 and 101.6 cm dbh (FLDSZCD = 4), and 10% with trees >101.6 cm dbh (FLDSZCD = 5), only the ages of trees in size class 3 (the predominant size class) would be used to determine FIA stand age. The same stand in interior states would have a mean age calculated from all trees >22.9 cm dbh.

Ages of individual trees within the stand-size class are usually obtained via ring counts on increment cores taken at breast height (e.g., from among trees cored to calculate the site index, or to meet the requirement of at least one cored tree per crown class (coastal) or diameter class (interior) per species per condition) [54]. Increment cores are generally ring-counted in the field, not crossdated using standard higher-accuracy dendrochronological techniques [55]. FIA stand age is then estimated or calculated as an average of the ages of dominant and codominant crown class trees sampled in this fashion, either unweighted or weighted by the relative abundance of tree sizes within the stand-size class [54]. In all states, stand age is sometimes based instead on documentary evidence or expert judgment that considers site productivity and tree diameters (for example, when coreable trees are lacking).

### Hypothesis 1: Trees older than FIA stand age should be rare

To investigate the distribution of live trees significantly older than the FIA stand age (hereafter “older trees”) within FIA plots, we obtained plot lists for the same plots analyzed in Odion et al. [46] (courtesy C. T. Hanson), which had been selected from wilderness or roadless areas within six distinct regions (Table 1). We used a subset of the same plots used in Odion et al. [46], selecting those plots that had 1) An associated tree list (an entry for the plot in the FIA TREE database with diameters for all trees and ages for a subset of live trees), and 2) A reported FIA stand age > 0 years (Table 1). Consistent with the stated methods of Odion et al. [46], we also only analyzed plots with a single FIA stand age, where there was only one condition class for the entire plot. The mean number of live trees per plot was 25 in interior states and 34 in coastal states (where FIA plots sample a larger area).

**Table 1:** Proportion of FIA plots with a percentage of total plot basal area comprised of trees significantly older than the reported FIA stand age.

	Number of plots		Percentage of total plot basal area comprised of older trees^3^		
Region^1^	Odion et al. [46]^2^	Current analysis^2^	>30%	>10%	>0%
Sierra Nevada West	141	96	0.219	0.541	0.771
Klamath	155	140	0.279	0.614	0.814
Eastern Cascades and E. Sierra	80	68	0.301	0.559	0.794
Northern Rockies	1352	870	0.349	0.575	0.662
Central Rockies	186	100	0.180	0.420	0.510
Southwest	223	198	0.359	0.687	0.793
Total (all regions)	2137	1472	0.322	0.580	0.697

1: Regions as defined by Odion et al. [46]; first three regions represent coastal states inventoried by the Pacific Northwest Research Station, and the last three regions represent interior states inventoried by the Rocky Mountain Research Station.

2: Current analysis based on a subset of plots from Odion et al. [46] that met the following criteria: 1) Comprised of a single, forested condition 2) A treelist with diameters for all trees and ages for a subset of live trees, and 3) A reported FIA stand age > 0 years. The total number of original plots reported in Odion et al. [46] did not all meet these three criteria.

3: Values indicate proportion of plots within a region where the percentage of total plot basal area contributed by trees significantly older than the FIA stand age, as estimated by age-diameter relationships, exceeded the specified threshold. Total proportions for all regions are averages of individual regions, weighted by the number of plots per region.

Because only a fraction of trees on each plot are aged (mean 23–41% across the six regions studied), it is not possible to construct full age distributions for an FIA plot. Therefore, for all stocked plots with a stand age greater than 0, we evaluated the distribution of older trees within the plot based on diameter-age relationships within the plot. This method of imputing missing ages, though imprecise, is preferable to building diameter-age relationships across broader geographic regions where growing conditions are even more variable [56]. Odion et al. [46] report that “the within-plot standard deviation of the proportional difference among individual tree ages and stand age” (for individual trees within the stand-size class used to generate a given FIA stand age) across all plots in this dataset was 0.14 or 14% ([46]; pg. 7). Under the assumption that FIA stand age represents the approximate time since a stand-replacing disturbance, Odion et al. [46] thus expect that 95% of all trees that regenerated in response to that disturbance should have ages within 28% (two standard deviations) of the FIA stand age ([46]; pg. 7). By this reasoning, trees with ages >1.28 times the FIA stand ages would be “significantly older” than the FIA stand age and unlikely to have regenerated following stand-replacing disturbance.

If FIA stand age truly reflects the timing of a high-severity fire event, then modern-day live trees that are older than the stand age must have survived that hypothetical high-severity fire. Our goal was to estimate, for each FIA plot, the proportion of the plot’s total basal area comprised of trees “significantly older” than the FIA stand age (tree ages >1.28 times the FIA stand age; hereafter “older” trees). Because not all trees within an FIA plot are aged, we identified “older” trees among the un-aged subset of trees according to the following rule set. If a plot had at least 2 aged trees within 28% of the FIA stand age (following the Odion et al. [46] estimate of tree age variability described above), we identified this subset of trees as “*Trees*.*SA*”. We calculated the minimum diameter for un-aged trees likely to be older than the FIA stand age (“*d*.*min*”) for that plot as the mean diameter of *Trees*.*SA* plus two standard deviations. If a plot had only 1 aged tree within 28% of the FIA stand age (*Trees*.*SA* = 1; 8% of all plots), we built a regression model of *d*.*min* conditional on the mean diameter of *Trees*.*SA* for all plots within that region where *Trees*.*SA* > 1, and set *d*.*min* for that plot as the regression-predicted value of *d*.*min* for the diameter of the lone aged tree. Thus for all plots where *Trees*.*SA*>0, we classified “older” trees as those trees with diameters > *d*.*min*. For plots where no aged trees were within 28% of the FIA stand age (*Trees*.*SA* = 0; 6% of all plots), we only classified aged trees with ages >1.28 times the FIA stand age as “older” trees. Having thus classified “older” trees for all plots, we estimated the fraction of the total plot basal area comprised of these “older” trees, scaling the basal area of individual trees measured on different nested plot sizes to a per-hectare basis using TPA_UNADJ in the FIA database.

Our rules for identifying “older” trees (above) may underestimate the true number of older trees. We included trees from size classes larger than the predominant stand-size class in our estimate of *Trees*.*SA*. For example, if the stand size class was comprised of trees between 22.9 and 50.8 cm dbh, but an aged tree > 50.8 cm dbh had an age within 28% of the FIA stand age, we included that tree in our calculation of *d*.*min*. This inclusion of aged trees from larger size classes in *Trees*.*SA* increased the value of *d*.*min* and allowed for the possibility that some additional larger un-aged trees on the site could have regenerated following a stand-replacing disturbance during the year implied by the FIA stand age (the year implied by the FIA stand age is equivalent to [plot inventory year]–[FIA stand age]). Additional “older trees” that were alive during the year implied by the FIA stand age could also have died in the intervening years, further reducing our estimates of older tree contributions to plot basal area. The basal area of the surviving older trees would have increased in the decades between the year implied by the FIA stand age and the measurement date, thus potentially overestimating their past contribution to the stand basal area in the year implied by the FIA stand age. However, total stand basal area in many ponderosa pine and mixed-conifer forests has also increased relative to historical estimates due to a century of fire suppression, albeit not to the extent that tree density has increased [7]. In light of this and the conservative rules we used in defining older trees, we believe that our calculations are reasonable approximations of the true contributions of trees older than the FIA stand age to the stand basal area in the year implied by the FIA stand age.

We identified three important thresholds for the basal area fraction of older trees within a plot. Plots with 30% or more of total basal area comprised of older trees were considered evidence that high severity fire at the time of the stand age could not have occurred in the plot in question, because 70% basal area mortality is the minimum threshold for high severity fire as described by Odion et al. [46]. Plots with 10–30% of total basal area comprised of older trees were unlikely to have burned at high severity, because contemporary data show that 70–90% basal area mortality within patches of high-severity fire at the scale of an FIA plot (0.067–0.4 ha, depending on the state) is uncommon relative to >90% basal area mortality ([57]; J. Miller unpublished data; see Discussion). Conservatively, plots with <10% of basal area of trees significantly older than the FIA stand age, or plots with no trees significantly older than the FIA stand age, could hypothetically characterize areas resulting from high-severity fire. However as we note below, other mechanisms besides high-severity fire can also produce such stand structures.

### Hypothesis 2: Minimal tree recruitment in the absence of high-severity fire

To investigate the relationship between fire severity, tree recruitment and FIA stand age, we combined data from FIA inventory plots and nearby fire history studies from the International Multiproxy Paleofire Database (IMPD; ncdc.noaa.gov/data-access/paleoclimatology-data/datasets/fire-history). First, we selected FIA plot data according to the rules described in Odion et al. [46] to restrict analysis to plots within wilderness, National Park, or inventoried roadless areas that were comprised of a single, forested condition class. Departing from the rule set used by Odion et al. [46], we included all forest types meeting these criteria, rather than just ponderosa pine and mixed-conifer, to examine a broader range of possible fire regimes. Then, we identified IMPD study sites that had fire scar records as part of their data. We selected those FIA plots that were within 1-km of the center of an IMPD site and that met two criteria: stand age ≥80 years and ≥ 4 aged trees within the plot. We then calculated the year associated with the stand age and with each individual tree age by subtracting these ages from the inventory year attribute associated with the FIA plot visit, and compared individual tree establishment events and the stand age against the fire history record.

We also examined recruitment patterns from a set of plots with size and age data for all trees, from a collection of published studies [16, 19, 39, 58]. All plots ranged from 0.1–0.5 ha, so are on a comparable scale with FIA plot sample areas, and all plots had fire scar records (Table A in S1 File). We calculated the stand size class for these plots based on their dbh distributions, following FIA size class guidelines, and then averaged the ages of all trees within the stand size class to estimate a stand age value analogous to FIA plots. We did not select these plots to make inferences to the larger landscape, but simply to illustrate the range of stand age structures that can be generated in frequent-fire forests.

## Results

### Hypothesis 1: Trees older than FIA stand age should be rare

**Fig 1:**
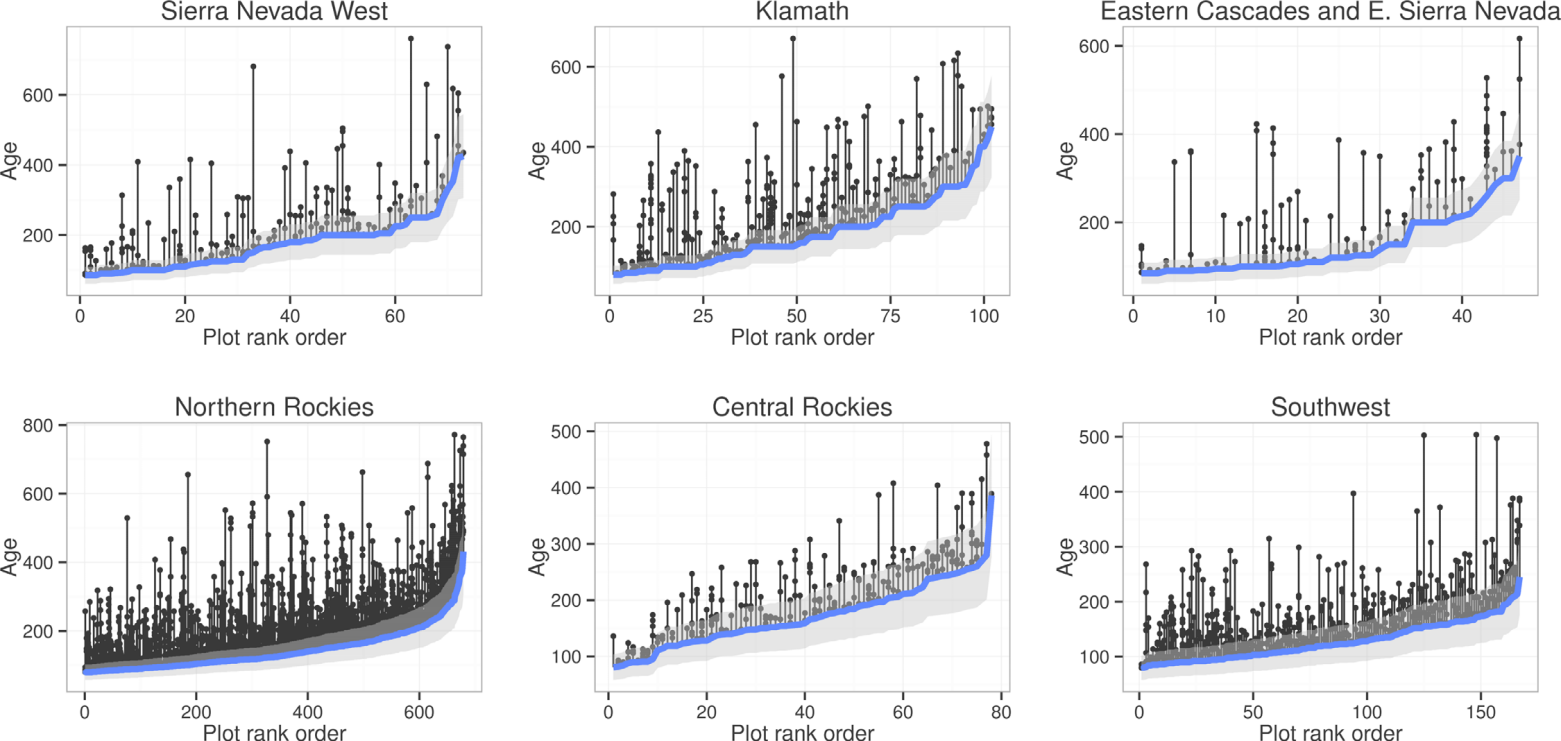
Ages of all individual cored trees that were older than their FIA stand age value. Data are arranged by plots in rank order of stand age (among plots with FIA stand age ≥80) to illustrate distribution of older tree ages within each plot. The height of the bottom blue line indicates the FIA stand age of plots, with a 95% confidence interval for individual tree ages based on the estimate of within-plot standard deviation of the proportional difference among individual tree ages and stand age of 0.14 (+/- 28% of FIA stand age; [46]). Each vertical line indicates a unique FIA plot and each point along that line indicates the age of a tree older than the reported stand age.

**Fig 2:**
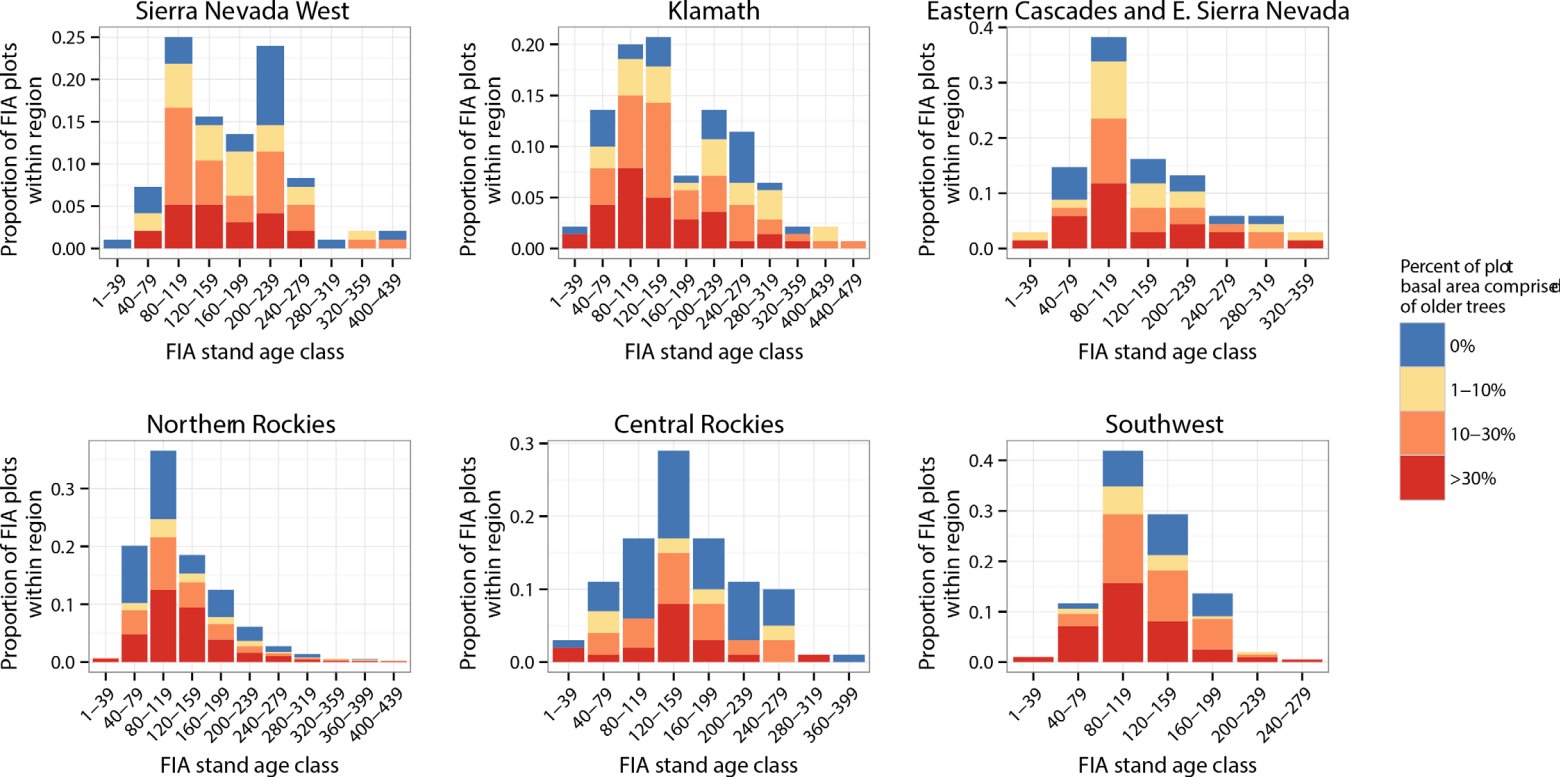
Distribution of basal area fraction in older trees, relative to stand age in plots sampled by Odion et al [46]. Older trees are defined as either aged trees older than 1.28 times the reported FIA stand age, or unaged trees with diameter greater than the mean diameter of aged trees within 28% of the FIA stand age plus 2 standard deviations. Note that vertical axis scaling varies among panels.

Individual tree ages within FIA plots with a single stand age were highly variable around the reported stand age. Many trees were considerably older than the FIA stand age (Fig 1). These older trees contributed substantially to the total stand volume (Fig 2). 32% of all the plots analyzed by Odion et al. [46] with an FIA stand age greater than 0 had more than 30% of their basal area in trees older than the stand age (based on age-diameter relationships within the plot), ranging from 18% of plots in the central Rocky Mountains to 36% in the southwest (Table 1). Furthermore, 58% of plots had more than 10% of their basal area in older trees, ranging from 42% in the central Rocky Mountains to 69% in the southwest (Table 1). Among all plots with an FIA stand age between 119 and 200 years old (the peak period of high-severity fire suggested by Odion et al. [46]), the proportion of plots with >30% and > 10% of their basal area in trees older than the FIA stand age increased to 36% and 63%, respectively.

### Hypothesis 2: Minimal tree recruitment in the absence of high-severity fire

**Fig 3:**
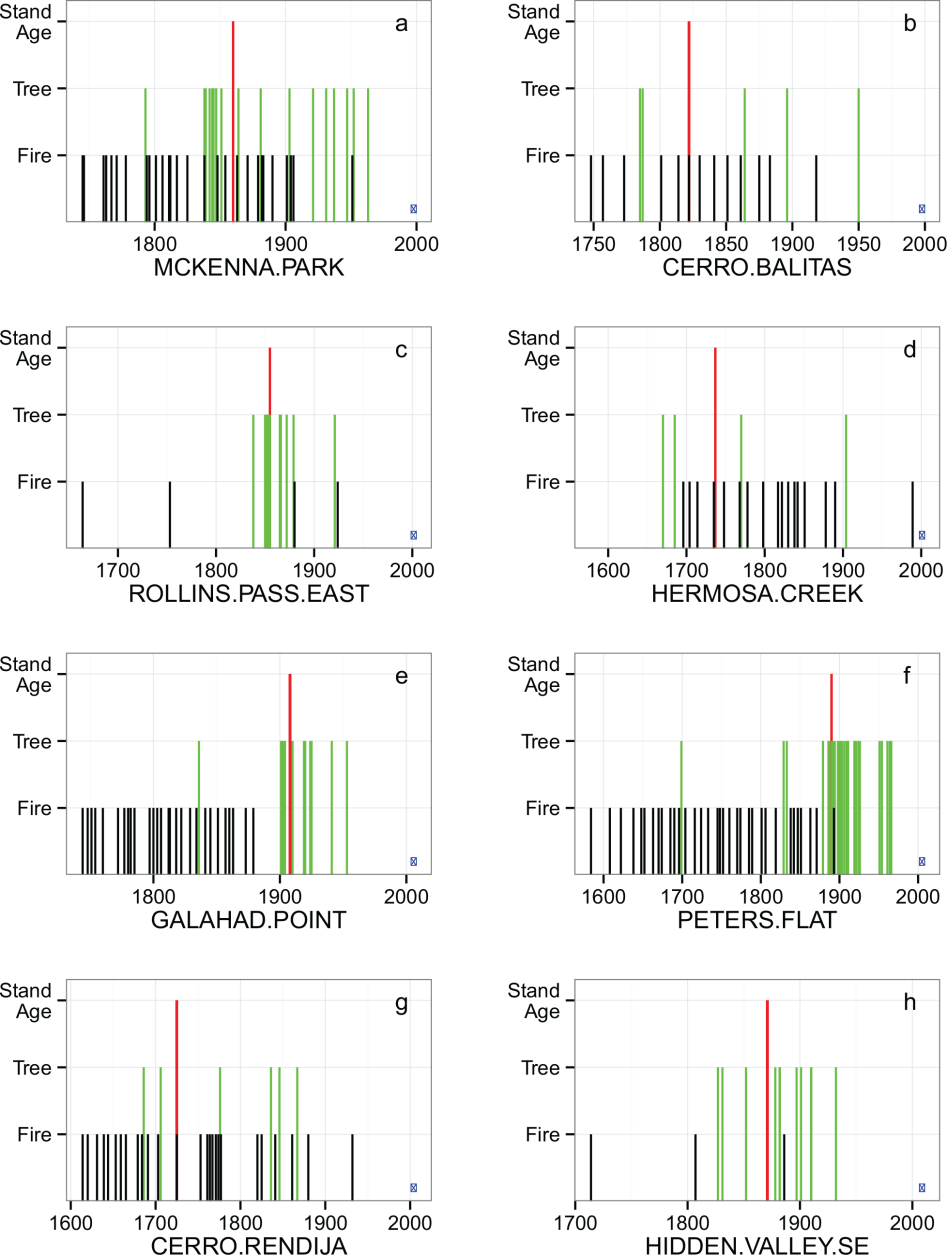
FIA plot overlay on fire history data. Red line indicates FIA stand age, green lines indicate individual tree ages, and black lines indicate fire occurrences based on fire scar data. Blue dot indicates FIA inventory year. McKenna Park, Cerro Balitas and Cerro Rendija sites were in New Mexico; Rollins Pass, Hermosa Creek and Hidden Valley sites were in Colorado; Galahad Point and Peters Flat sites were in Arizona.

Examining FIA data in the context of local fire history information reveals that individual tree recruitment events, and associated stand ages, are not consistently linked to high-severity fire events (Fig 3). Of the 8 fire history sites that fit our selection criteria (within 1-km of IMPD sites, stand age ≥80 years and ≥ 4 aged trees within the plot), only one (Rollins Pass East; Fig 3C) had an age distribution and fire history suggesting recruitment could be tied to a high severity event. This site, a subalpine forest in central Colorado, had a single fire event around 1880 after more than 130 years without fire, and nearby FIA data indicated a pulse of recruitment around this time, coincident with the “stand initiation year” implied by FIA stand age. Here it is conceivable that the recruitment pulse followed the fire event, although recruitment appears to pre-date the fire event; the absence of individual very old trees further supports this interpretation.

At all other sites, alternative explanations for individual tree recruitment events are likely. Two sites (Galahad Point and Peters Flat; Fig 3E and 3F) have high recruitment episodes and FIA stand ages dating to the initiation of fire suppression where fire had previously been frequent. In the case of Galahad Point, there is no evidence of fire within 20 years of the major recruitment event, and in both cases pre-suppression fire return intervals were extremely short. In three other cases, (Cerro Balitas, Hermosa Creek and Cerro Rendija), tree recruitment events appear to have occurred over a wide range of time within a landscape impacted by multiple fires, based on the distribution of ages of the limited number of aged trees within the plot (Fig 3B, 3D and 3G). At these three sites, none of the aged trees are within 25 years of the FIA stand age value. In McKenna Park, a pulse of recruitment occurred in the early 1800’s after more than 10 fires during the previous century. While it is conceivable that the fire in 1837 could have burned at high-severity in some places in McKenna Park, none of the 11 fire-scarred trees that recorded the 1837 fire at that study site died as a result of that fire. Five other fire scarred trees at that site also established well before 1837 and also survived that event, and the 16 fire scarred trees sampled there also survived dozens of previous and subsequent fires in that stand [59]. The post-1837 recruitment pulse is more likely attributable to the combination of fire-free periods coinciding with favorable weather conditions (e.g., see [59], and Discussion below).

Age data from forest plots in other published studies with known fire history records from fire scars collected within or adjacent to the plot illustrate a wide range of stand conditions and possible stand ages, similar to the FIA plots described above. Some stands are dominated by older trees with very few younger trees, and their stand age estimates are generally >250 years (e.g. Figure A panels a, i in S1 File). Other stands have recruitment events and estimated stand ages that fall during long fire-free periods (e.g. Figure A panels e, h in S1 File). Still others have stand ages < 200 years old due to recruitment pulses during the 1800’s, despite a long record of frequent fire from fire scars and the presence of numerous older trees co-occurring within the plot (e.g. Figure A panels d, k, l in S1 File). The uneven age structure in all these plots make it unlikely that any particular stand age estimate represents the date of a high-severity fire.

## Discussion

### Use of FIA data to sample historical disturbance regimes

Contemporary forest plot data from the FIA network and other sources provide a wealth of information on contemporary forest dynamics. For example, FIA data have been used successfully to shed light on forest carbon dynamics [60], characterize fire hazard at the landscape scale and the costs of fuel treatments [61] and to detect evidence of tree species shifts in response to climate change [62]. However, contemporary forest plot data are of limited use for drawing conclusions about the historical extent and rotation of high-severity fire in uneven-aged forests, particularly when based on averages of a subset of tree ages in a stand and without physical evidence of past fire events. As we have shown, the FIA stand age variable alone does not adequately account for the abundance of older trees within the plot, nor does stand age require that tree establishment be coupled with a stand-replacing disturbance event. FIA stand age does not support inferences about the drivers of demographic variability, including mortality and recruitment, both of which have complex causation in frequent-fire, ponderosa pine and mixed-conifer forests. Indeed, a single estimated stand age cannot reflect the multi-cohort structure of uneven-aged stands, no matter how many individual trees are cored. Thus, this simplified attribute cannot be used to estimate a stand replacing disturbance date, a time since fire distribution, or be the basis for calculating high-severity fire rotation.

The abundance of plots with trees older than the FIA stand age suggest that the extent of high-severity fire identified by Odion et al. [46] was overestimated across the sampled area. We found that among plots analyzed by Odion et al. [46] with reported FIA stand ages >0 years, trees older than the FIA stand age accounted for more than 10% of plot basal area in 58% of plots, indicating that that an interpretation of high-severity fire during the year implied by the FIA stand age is unlikely for nearly 60% of the plots analyzed (see “Quantifying high severity fire in ‘mixed-severity’ regimes“below). Furthermore, several sources of uncertainty bias our findings towards underestimating the plot basal area fraction comprised of trees older than the FIA stand age. First, in plots where few aged trees fell within 28% of the stand age and those trees had a wide range of diameters, the resulting critical diameter threshold (2 standard deviations above the mean diameter) was larger than the maximum diameter of any individual tree within 28% of the stand age. Thus, plots with smaller and more variable sample sizes had a stricter standard for classifying a tree as “older”. Second, for the 6% of all plots where no aged trees were within 28% of the FIA stand age, we only classified a small number of aged trees as “older” (those cored trees that were >1.28 times the FIA stand age), thus excluding a number of large trees that were likely older than the FIA stand age. Third, the use of the 28% range around the FIA stand age for identifying trees that regenerated following a stand-replacing disturbance assumes that the hypothetical disturbance happened at the early end of this range for all plots. Collectively, this suggests that our estimates of trees older than the proposed high-severity fire date at a given FIA plot (Fig 2) are low.

High-severity fire is somewhat more plausible in the remaining 42% of FIA plots where <10% of the stand basal area is comprised of trees older than the stand age, but even in these plots, the scarcity of older trees is not necessarily an indicator of past high-severity fire. For example, given the small FIA plot size in interior states (1/15th ha), and low density of very large trees in some reconstructed frequent-fire forests (e.g. trees > 90 cm dbh present at densities of <15 trees ha^-1^ [7, 33, 63]), very large trees may therefore be completely absent from the plot by chance alone. The small plot size may be part of the reason why FIA plots from interior states in the northern and central Rockies have a higher proportion of plots with 0% of the basal area in older trees than coastal states (Table 1). However, dry forests in these regions may also experience more high-severity fire than the other regions examined [12]. In coastal states, where FIA plots are 0.4 ha, low-density features such as very old large trees (e.g. >300 years) are more likely to be sampled than they are on FIA plots in interior states, but may still comprise a small fraction of the stand basal area relative to younger trees that comprise the predominant stand size class used to generate FIA stand age. This could explain the greater abundances of plots with 1–10% of their basal area in older trees in coastal states relative to interior states (Table 1).

Our analysis of FIA plots in conjunction with fire history data supports the conclusion that the FIA stand age attribute is not closely linked to likely stand-replacing fire events (Fig 3). The location coordinates of FIA plots in the public database are offset from their true location by a random value of <1 km, in order to maintain privacy on privately-owned lands and protect FIA plots from being managed differently than the larger landscape that they are sampling. Therefore the true location of the FIA plots in our analysis may be closer to or farther from the center of the fire history study, which does introduce some uncertainty to the analysis (although the total area surveyed by fire history studies to determine consensus fire-scar dates is variable and not always well documented). Nonetheless, the fire history studies analyzed here are used to give an idea of the general fire regime in an area, not to represent the definitive fire history of an FIA plot. Additional FIA plots could be included to expand the sample size if the 1km buffer around the fire history centroids were expanded, but this would come at a cost of further reducing the confidence that the historical fire regime at an FIA plot is captured by the fire history data.

The expectation of Odion et al. ([46]; pg. 4) was that forests experiencing predominantly low-moderate severity fire should be “dominated by older age classes”; in other words, they imply that plots in the 200–400 year old FIA stand age classes should be more abundant than plots with FIA stand ages <200 years (though this breakpoint is rather arbitrary). Although individual trees can reach these older ages, there are numerous reasons why 100–200 year old trees might numerically dominate contemporary western ponderosa pine and mixed-conifer forests. Many frequent-fire forests historically had very low densities and basal areas of old trees [7, 19, 33, 37, 52, 64–67]. Compounding these historically low densities is a well-documented trend towards increased mortality of large trees during the 20^th^ century, coincident with a period of increased temperatures and water deficit [68, 69]. Insect outbreaks have caused large-scale mortality events of canopy-dominant trees prior to the onset of fire suppression [70], and bark beetles often target the largest trees within a stand [71, 72]. Some wilderness areas and National Parks were selectively logged prior to their designation as protected regions [7, 69, 73], with large trees targeted for removal. In addition, gradual attrition of larger trees with frequent low- severity fire can still lead to considerable overstory mortality over time, especially when combined with other stressors [74].

The “younger”, 100–200 year old trees that today are numerically dominant in the canopy of some FIA plots did not necessarily require high-severity fire events to establish. Trees may have established during fire-free intervals (e.g. 10–30 years; Fig 3) during the century prior to the implementation of federal fire suppression policy of the early 1900’s. In many places, fire frequency began to decline during the 1800’s with the decline of Native American populations following the westward expansion of Euro-American settlement [75, 76]. Extended fire-free periods during the early 1800’s have been documented across western North America, which coincide with intensive grazing across the west [77] and increased intensity of El Niño cycles [40, 41, 78]. These wetter periods that drove down fire frequencies also likely created favorable conditions for recruitment; the cohorts of trees that established during this period could have survived recurring low-severity fires for another 50–100 years to dominate the canopy in many contemporary mixed-conifer forests across the western United States [39–41, 79, 80]. This is the likely explanation for the forest structure at McKenna Park (Fig 3A), where the dominant cohort of trees established during the period from 1838–1847, which was one of the wettest and longest fire-free periods of the 300 years prior to the modern fire-suppression era [41, 59, 79].

Clearly, the factors driving the present-day age structure in any given FIA plot are complex in uneven-aged forests, and cannot be attributed to a single cause such as high-severity fire across thousands of FIA plots.

### Quantifying high severity fire in “mixed-severity” regimes

The broader question is how to best describe the historical scale and frequency of high-severity fire in forests where high-severity fire occurs but does not predominate. The increasing use of the term “mixed-severity” to describe fire regimes across broad geographical regions and forest types [46, 81–84] warrants further scrutiny. “Mixed-severity” fire regimes are often defined as including low-, moderate- and high-severity effects. However, without an explicit description of the size of high-severity patches, “mixed-severity” fire regimes can be interpreted in many ways, because at different scales, all fires have some proportion of low, moderate and high-severity effects [16, 30]. Odion et al. ([46]; pg. 4) acknowledge this point, but at the same time argue, “a fire regime with a high-severity component *of any amount* should not be classified as low/moderate-severity” ([46], pg. 12; emphasis added). This is not consistent with the literature on how fire regimes are described or classified [2], nor is it logical: by extension, there can be no “high-severity” fire where even a single tree survives, and there can be no “low/moderate-severity fire” where even a single tree is killed. Even in very frequent fire systems (return interval ≤ 20 years) where low- to moderate-severity effects predominate, groups of trees can experience high-severity fire, with high-severity patch sizes ranging from 0.4 to >50 ha, due to the heterogeneity of fire effects based on local variation in topography, fuels, weather, and prior fire effects [7, 31, 85, 86]. Under a general definition of “mixed-severity” fire, a landscape that experiences low-moderate severity fire with several 0.4–50 ha patches of high-severity would be called “mixed-severity”, however the same landscape with predominantly high-severity effects containing several 0.4–50 ha patches of low-moderate severity could also be termed “mixed-severity”. Therefore, this general use of the “mixed-severity” classification is not informative, because the size of historical high-severity patches at a landscape scale is a crucial metric to consider when assessing departure from historical fire effects, especially in relation to the large patches of high-severity fire with complete canopy mortality (e.g. >1000 ha) that have occurred in recent fires [8–10, 48, 57, 87].

Odion et al. [46] claim that evidence of high-severity fire at the FIA plot scale based on stand age represents a statistical sample of the most recent high-severity fire at roughly the same time and mortality level across a much larger area (e.g. their entire dataset represents “a sample population of about 5.1 million ha of unmanaged” forest [pg. 7]). Implicit in this extrapolation to larger spatial scales is that FIA plots are samples of larger high-severity fire patches that occurred at the time of the FIA stand age. However, thresholds of mortality commonly used to distinguish fire severity classes at the landscape scale (e.g. 70%) should not be applied to the FIA plot scale (0.067–0.4 ha), because of the principles of fire behavior that drive high-severity fire patches. High-severity fire generally occurs when surface fuels are very high, and/or when ladder fuels form a continuous profile from the surface to the canopy [4], producing crown fires and/or high heat loads that kill the vast majority (>>90%) of trees within the high-severity patch. The minimum threshold of 70% mortality used by Odion et al. [46] to describe a high-severity patch (and the 75% threshold employed by Landfire) was not developed to describe mortality within a stand, but rather mortality across an entire fire [2, 88].

Modern data on mortality in high-severity patches illustrate the importance of scale in the interpretation of fire effects. A modern burn severity classification scheme that relies on the relative difference in normalized burn ratio (RdNBR) [89], classifies post-fire Landsat 30m pixels as “high-severity” if the change from pre-fire photosynthetic activity of the pixel exceeds a threshold such as 75% or 90%. A “high-severity patch” at the landscape scale can then be defined as adjacent pixels sharing a high-severity classification. However, extensive ground-truthing of “high-severity” patches, even those derived from a 75% threshold, reveals that live basal area within a high-severity patch is 0 m^2^ ha^-1^ in over 80% of the hundreds of plots surveyed (plots range in size from 0.07 ha to 0.63 ha), and is greater than 10 m^2^ ha^-1^ in fewer than 10% of plots located away from the edge of the high-severity patch [57]. In other words, sub-ha scale plots wherein 70–99% of tree basal area died are uncommon as a proportion of total area burned in a given fire, especially if they are near the middle of a large high-severity patch [57]. Therefore, if FIA stand age data are to be interpreted as samples of high-severity patches that were much larger than the plot scale (e.g. > 5 ha high-severity patches), then the majority of plots should have 0% of their basal area in older trees (i.e. be even-aged), which we demonstrate clearly is not the case (Fig 2).

Odion et al. [46] present their analysis of FIA stand age data in the context of a broader summary of literature that they argue supports the existence of “significant amounts of weather-driven, high-severity fire” in ponderosa pine and dry mixed-conifer forests ([46]; pg. 1). A treatment of the literature cited to support this claim is beyond the scope of this paper and has been responded to elsewhere [7, 56, 64, 90]. However, we emphasize that there are literally hundreds of studies over the past century, drawing on diverse methods including primary source accounts, historical reconstructions, dendrochronological field and laboratory-based analyses, climatological data, modern reference forests, and principles of fire behavior, which support the theory that most ponderosa pine forests and many low-mid elevation mixed-conifer forests were generally heterogeneous, low-density, uneven-aged forests that experienced frequent, fuel-limited, primarily low-moderate severity fire regimes [2–4, 6–8, 16, 17, 19, 20, 33, 37, 41, 43, 47, 50–52, 58, 63, 64, 79, 80, 91–107].

High-severity patches did occur in these forests, as many of the above studies attest. However, the pertinent question is, if a fire regime is to be considered mixed-severity, how big were the high-severity patches historically, how often did they occur, and how have those parameters changed in current forests? Identifying cohorts of even-aged trees is a viable way to quantify potential historical high-severity patches [6, 19, 33, 45]; however, in light of the issues with average-age data we have discussed, care must be taken to accurately define the spread of ages within, and spatial extent of, these patches. Other possible mechanisms (such as fire-free intervals) must also be tested equally.

We highlight four guidelines for future efforts to infer historical fire severity from age-structure data: 1) Complete age structures within the putative even-age patches must be developed, in order to identify the presence and abundance of older trees within the cohort. 2) When possible, evidence of fire occurrence at the time of cohort initiation (e.g. fire scars, fire-killed tree death dates) should be obtained from trees elsewhere on the landscape outside the even-aged patch. 3) If inference is to be made regarding high-severity patch size, sampling must be conducted across an area greater than the even-aged cohort, and included in the complete age structure data. 4) If inference is to be made regarding the rotation or return interval of high-severity patches, a large area of landscape (in relation to the size of even-aged cohorts) must be sampled. We recognize that this is not trivial work, but the FIA database is not a suitable alternative to this approach, despite its convenience and accessibility. The FIA stand age variable (or any single estimate of stand age from uneven-aged forests) cannot be used to characterize fire history in uneven-aged forests typical of western North America, primarily because 1) it does not account for the abundance of older trees or the diverse and complex age structure within the plot or the surrounding stand, and 2) it does not adjudicate whether trees recruited following high-severity disturbance, or responded to some other mechanism driving forest demography.

## Supporting Information

S1 File Additional age structure plots with fire history Illustration of the range of age structure and possible stand age values associated with frequent fire regimes (Figure A). Black lines represent fire years and green lines represent tree establishment dates (= sample date–breast height age–correction factor [5 years a-h, 8 years i-l]). Red vertical lines represent stand age estimated by averaging all tree ages within the predominant size class for the plot. Blue point represents year measurements were taken. Plot information and size class data is provided (**Table A**).
(DOCX)
