# Supplementary material for: Average Stand Age from Forest Inventory Plots Does Not Describe Historical Fire Regimes in Ponderosa Pine and Mixed-Conifer Forests of Western North America
Source: PLoS One. 2016 May 19;11(5):e0147688. doi: 10.1371/journal.pone.0147688 (PMC4873010; doi:10.1371/journal.pone.0147688)
Supplement: S1 File — Black lines represent fire years and green lines represent tree establishment dates (= sample date–breast height age–correction factor [5 years a-h, 8 years i-l]). Red vertical lines represent stand age estimated by averaging all tree ages within the predominant size class for the plot. Blue point represents year measurements were taken. Plot information and size class data is provided (Table A). (DOCX) [file pone.0147688.s001.docx]

**Figure A**

**Table A**: Plot information from age structure plots

| Site | State | Plot Size (ha) ^1^ | Minimum dbh (cm) ^1^ | Stand Size Class Code ^2^ | Field Size Class Code ^3^ | Source |
| --- | --- | --- | --- | --- | --- | --- |
| Jemez Mountains | NM | 0.1 (0.01) | 20 (2.5) | 3 | NA | (Falk, 2006) (Falk, unpublished data) |
| Mt Rushmore | SD | 0.12-0.17 | 20 | 3 | NA | (Brown et al., 2008) |
| Archuleta Mesa | CO | 0.21-0.5 | 20 | 3 | NA | (Brown and Wu, 2005) |
| Big Oak Flat | CA | 0.1 (0.025) | 30 (10) | - | - | (Scholl and Taylor, 2010) |
| BOF-54 |  |  |  | NA | 5 |  |
| BOF-56 |  |  |  | NA | 4 |  |
| BOF-98 |  |  |  | NA | 3 |  |
| BOF-81 |  |  |  | NA | 4 |  |

1: Number in parentheses indicates nested plot size for smaller trees, and minimum dbh for these smaller trees.

2: Stand size class 3, estimated by FIA for interior states, represents trees > 22.9 cm dbh

3: Field size class, estimated by FIA for coastal states, represents trees 22.9 cm – 50.8 cm dbh (size class 3), 50.8 cm – 101.6 cm dbh (size class 4), or > 101.6 cm dbh (size class 5).
